# Joint Sampling and Transmission Policies for Minimizing Cost Under Age of Information Constraints †

**DOI:** 10.3390/e26121018

**Published:** 2024-11-25

**Authors:** Emmanouil Fountoulakis, Marian Codreanu, Anthony Ephremides, Nikolaos Pappas

**Affiliations:** 1 Ericsson, 16483 Stockholm, Sweden; 2 Department of Science and Technology, Linköping University, 60174 Norrköping, Sweden; marian.codreanu@liu.se; 3 Department of Electrical and Computer Engineering, University of Maryland, College Park, MD 20742, USA; etony@umd.edu; 4 Department of Computer and Information Science, Linköping University, 60174 Linköping, Sweden; nikolaos.pappas@liu.com

**Keywords:** age of information (AoI), Lyapunov optimization, randomized policies, scheduling, wireless networks

## Abstract

In this work, we consider the problem of jointly minimizing the average cost of sampling and transmitting status updates by users over a wireless channel subject to average Age of Information (AoI) constraints. Errors in the transmission may occur and a policy has to decide if the users sample a new packet or attempt to retransmission the packet sampled previously. The cost consists of both sampling and transmission costs. The sampling of a new packet after a failure imposes an additional cost on the system. We formulate a stochastic optimization problem with the average cost in the objective under average AoI constraints. To solve this problem, we propose three scheduling policies: (a) a dynamic policy, which is centralized and requires full knowledge of the state of the system and (b) two stationary randomized policies that require no knowledge of the state of the system. We utilize tools from Lyapunov optimization theory and Discrete-Time Markov Chain (DTMC) to provide the dynamic policy and the randomized ones, respectively. Simulation results show the importance of providing the option to transmit an old packet in order to minimize the total average cost.

## 1. Introduction

The Age of Information (AoI) is a metric that captures the timeliness or freshness of the data [1,2,3]. It was first introduced in [4], and it is defined as the time elapsed since the generation of the status update that was most recently received by a destination. AoI can play an important role in applications with freshness-sensitive data, e.g., environment monitoring, smart agriculture, sensor networks, etc. Consider a cyber–physical system, where a number of sensors sample and transmit freshness-sensitive data (e.g., temperature, humidity, solar radiation level) to a destination over a wireless channel. However, devices that sample fresh information could perform more sophisticated tasks rather than simply sample new information for transmitting it to the destination. For example, an Internet of Things (IoT) device can perform initial feature extraction and pre-classification by using machine learning tools. In such cases, the sampling cost cannot be ignored, especially for low-power budget wireless devices. In this work, we consider a set of users who sample and transmit fresh information over error-prone channels. Our goal is to provide scheduling policies that minimize the transmission and sampling cost of the communications system while satisfying the average AoI requirements. We address the trade-off between average AoI and total cost. Simulation results show for which cases it is beneficial to send an old yet not stale packet to minimize the total cost while providing the required freshness of the data at the destination.

### 1.1. Related Works

The average and peak AoI analysis in queueing systems has been extensively studied over the last few years [5,6,7]. The studies mainly focus on the derivation of closed-form or approximated expressions of the average and peak AoI in different network set-ups and under different queueing disciplines. Also, AoI performance analysis has been studied in random-access networks [8,9], as well as in a CSMA environment [10]. In [8,9], the authors study the interplay between AoI-oriented users and delay-constrained users in random-access networks.

Optimization and control of wireless networks with AoI-oriented users has been recently investigated by the research community for a plethora of network scenarios [11,12,13,14,15,16,17,18,19,20,21,22,23,24]. The authors in [11,12,13] study single-hop networks with stochastic arrivals, in which the status updates randomly arrive to the users’ queues and wait to be transmitted to the destination. Max-weight policies, as well as stationary randomized policies, are provided for minimizing the AoI. On the other hand, scenarios, in which the *generate-at-will* policy is utilized, have been considered in the literature [15,16,17,18,19,20,21,22]. In these scenarios, which are closer to our work, no random arrivals are considered, and the transmitters generate status updates from a source based on their or scheduler’s decisions. In [22], the problem of AoI minimization with throughput constraints in a wireless network with multiple users is considered. The authors provide a lower bound for the average AoI. Several scheduling policies for minimizing the AoI are proposed. Furthermore, the AoI minimization problem with heterogeneous traffic has been studied in [15,25], and the power minimization under AoI constraints in [16]. General non-decreasing cost functions of AoI have been considered for the AoI minimization problem in [17]. In [23], the authors consider the joint sampling and scheduling problem for minimizing AoI in multi-source systems. In [24], the authors consider the AoI minimization problem with average energy consumption constraints of which the optimal policy is shown to be a randomized mixture two stationary deterministic policies.

In [18,19,20,21], the optimization of AoI in IoT and energy harvesting communication systems has been studied. In [18,19], the authors formulate the AoI minimization problem for scenarios, in which energy-harvesting nodes with finite battery capacity sample fresh information and transmit it to the destination. The problems are formulated as a Markov Decision Process (MDP) which are solved by using tools from dynamic programming and reinforcement learning. In [20,21], the authors consider the AoI minimization in scenarios in which IoT devices with heterogeneous traffic and limited energy budget, respectively, sample and transmit fresh information.

AoI has also been considered an important metric for remote estimation [26,27,28,29,30] and a first step towards semantics-aware communication systems [31,32,33]. In [26], the authors address the trade-off between reliability and freshness of information in a wireless sensor system. In [27], the metric of Age of Incorrect Information (AoII) is introduced for remote estimation. In [28], the authors consider continuous-time Markovian sources observed by a remote monitor. In [29], the authors design an optimal sampler for remote estimation of random processes by utilizing the framework of continuous MDP. In [30], the authors study the AoI as a performance metric for remote estimation in a wireless networked control system.

The scenario that is closer to our work is the one considered in [20]. The authors consider an IoT device sampling and sending information over a wireless fading channel to a receiver. Sampling, as well as the transmission cost, are considered, and the power transmission is adapted according to the channel conditions to ensure reliable communication. The authors formulate the AoI minimization problem with energy constraints as a Constrained Markov Decision Process (CMDP), and they propose a structural-aware optimal policy for solving the CMDP problem. In our work, the goal is different, namely the cost minimization under average AoI constraints. Furthermore, we consider error-prone wireless channels, an assumption that makes our problem fundamentally different. In our case, the transmitters also have the option to retransmit an old packet, and it is shown that this option can dramatically improve the system cost while guaranteeing the required data freshness at the receiver for each user.

### 1.2. Contributions

In this work, we consider the minimization of the total average cost while guaranteeing average AoI below a threshold for each user. We propose three scheduling policies. The first scheduling policy, named Drift-Plus-Penalty (DPP), is a dynamic policy that takes decisions in each slot to minimize the cost. However, the DPP policy requires full information, i.e., the cache state of each user, and the waiting time of the corresponding packet. For this reason, we propose two stationary randomized policies that require no information. The first stationary randomized policy, named Old-or-Fresh Randomized Policy (OFRP), allows the scheduler to probabilistically decide at every time slot the scheduled user. Then, the scheduled user decides the action by itself, i.e., either to sample and transmit, to transmit an old packet, or to remain idle. The second stationary policy, named Fresh-only Randomized Policy (FoRP), which is a simplified version of the first, allows the scheduler to probabilistically decide which user to schedule at every time slot. Then, the scheduled user decides either to sample and transmit or to remain idle.

The contributions of our work are summarized below:

- We propose a dynamic policy based on Lyapunov optimization, and we prove that its solution is near-optimal.
- We propose two stationary randomized policies. We model the system as two Discrete-Time Markov Chains (DTMCs), and we provide the expressions for the total average cost, the average AoI for each user, as well as, the distribution of the AoI.
- Simulations results are provided, and they show the performance of the proposed scheduling policies. Also, simulation results show how the option of transmitting an old packet can dramatically improve the total average cost.

## 2. System Model

**Figure 1:**
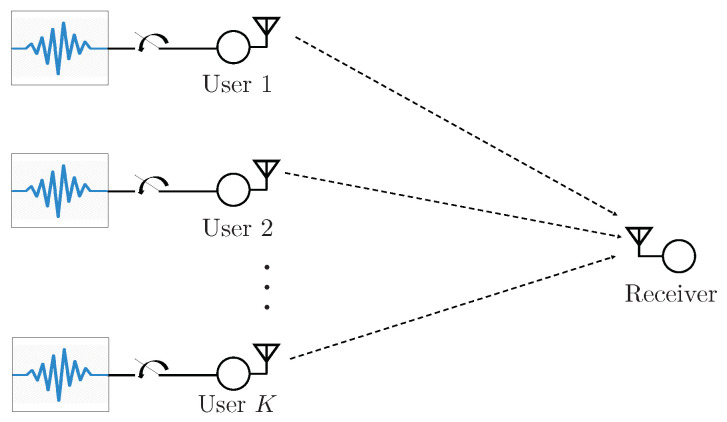
System model.

We consider a set of users, denoted by $K=\left\{1,\ldots ,K\right\}$, who sample fresh information and send this information, in form of packets, to a receiver over a wireless fading channel, as shown in Figure 1. Time is assumed to be slotted. Let $t\in Z_{+}$ be the *t*th slot. Note that due to fluctuations in the fading channel, we may have errors in the transmissions. Therefore, the packet is successfully transmitted to the receiver with some probability. Note that in the case of error transmission, the user keeps the packet in its cache for possible retransmission during the next slots.

We consider that at every time slot, up to one user is scheduled to transmit a packet. Let $p_{k}$ be the success transmission probability of user *k*. We denote by $Q_{k}\left(t\right)$ the state of the cache of user *k*. $Q_{k}\left(t\right)$ takes the value of 1, if there is a packet in the cache, and 0 otherwise. We denote by $s_{k}\left(t\right)$ the action of the user *k* to sample and transmit in time slot *t*, where

(1) $$\begin{array}{c} s_{k}\left(t\right)=\left\{\begin{array}{c} 1,\;\mathrm{if}\;\mathrm{the}\;\mathrm{user}\;k\;\mathrm{samples}\;\mathrm{and}\;\mathrm{transmits}\;\mathrm{in}\;\mathrm{time}\;\mathrm{slot}\;t,\\ 0,\;\mathrm{otherwise}. \end{array}\right. \end{array}$$

We denote by $\mu_{k}\left(t\right)$ the action of user *k* to transmit an old packet in time slot *t*, where

(2) $$\begin{array}{c} \mu_{k}\left(t\right)=\left\{\begin{array}{c} 1,\;\mathrm{if}\;\mathrm{the}\;\mathrm{user}\;k\;\mathrm{transmits}\;\mathrm{an}\;\mathrm{old}\;\mathrm{packet}\;\mathrm{in}\;\mathrm{time}\;\mathrm{slot}\;t,\\ 0,\;\mathrm{otherwise}. \end{array}\right. \end{array}$$

Note that $\mu_{k}\left(t\right)$ can take the value of one only if there is a packet in the cache. We also denote by $d_{k}\left(t\right)$ the successful packet transmission of user *k*, where

(3) $$\begin{array}{c} d_{k}\left(t\right)=\left\{\begin{array}{c} 1,\;\mathrm{if}\;\mathrm{the}\;\mathrm{receiver}\;\mathrm{receives}\;a\;\mathrm{packet}\;\mathrm{from}\;\mathrm{user}\;k\\ \;\mathrm{during}\;\mathrm{the}\;\mathrm{time}\;\mathrm{slot}\;t-1,\\ 0,\;\mathrm{otherwise}. \end{array}\right. \end{array}$$

Note that $d_{k}\left(t\right)$ takes the value of one, if $s_{k}\left(t-1\right)=1$ or $\mu_{k}\left(t-1\right)=1$, with probability $p_{k}$, and $s_{k}\left(t\right)+\mu_{k}\left(t\right)\leq 1,\;\forall k$. It follows that $E\left\{d_{k}\left(t\right)|\mu_{k}\left(t-1\right),s_{k}\left(t-1\right)\right\}=p_{k}\mu_{k}\left(t-1\right)+p_{k}s_{k}\left(t-1\right)$. By applying the law of iterated expectations, we obtain $E\left\{d_{k}\left(t\right)\right\}=p_{k}E\left\{\mu_{k}\left(t-1\right)\right\}+p_{k}E\left\{s_{k}\left(t-1\right)\right\}.$

### 2.1. Age of Information

The AoI represents how “fresh” is the information from the perspective of the receiver. Let $A_{k}\left(t\right)$ be a strictly positive integer that depicts the AoI associated with user *k* at the receiver. If the received packet has been sampled during slot *t* and its transmission is successful, then $A_{k}\left(t+1\right)=1$. AoI takes the value of one because the successful transmission takes one slot to be performed. On the other hand, if the received packet has been sampled during the previous slots, then the Age of Information also depends on the time of the packet waiting for successful transmission. In this case, $A_{k}\left(t+1\right)>1$. Therefore, the value of AoI depends on the waiting time of the packet in the cache of the corresponding user. Furthermore, we assume that the value of AoI is bounded by an arbitrarily large finite value $M\in Z_{+}$. This assumption is considered for two reasons: (1) In practical applications, values of AoI that are larger than a threshold will not provide us additional information about the staleness of the packet [18,20], (2) Assuming unbounded AoI will significantly complicate our analysis without giving additional insights into the performance of the system. Moreover, this assumption has been widely used in recent works that study the average AoI [34,35,36]. Let $A_{k}^{p}\left(t\right)$ represent the system time of the packet in queue *k*, i.e, the waiting time of the packet. By definition, we have $A_{k}^{p}\left(t\right)=t-\tau_{k}^{s}\left(t\right)$, where $\tau_{k}^{s}\left(t\right)$ is the most recent sampling time. Naturally, the value of $\tau_{k}^{s}\left(t\right)$ changes only if a new packet is sampled at the beginning of slot *t*. We consider that the decisions are taken at the beginning of each slot. In order to avoid having values of $A_{k}^{p}\left(t\right)$ that are greater than the AoI at the destination, we bound the value of $A_{k}^{p}\left(t\right)$ as $A_{k}^{p}\left(t\right)=\min\left\{t-\tau_{k}^{s}\left(t\right),M-1\right\},\;\forall k.$ Note that, when $A_{k}^{p}\left(t\right)$ reaches the value of M−1, the user drops the packet, and its cache becomes empty. We assume that a packet’s transmission takes one time slot to be performed. The evolution of the AoI at the receiver for user *k* is

(4) $$\begin{array}{c} A_{k}\left(t+1\right)=\left\{\begin{array}{c} A_{k}^{p}\left(t\right)+1,\;\mathrm{if}\;d_{k}\left(t+1\right)=1,\\ \min\left\{A_{k}\left(t\right)+1,M\right\},\;\mathrm{if}\;d_{k}\left(t+1\right)=0. \end{array}\right. \end{array}$$

If the received packet has been sampled in the slot *t*, then $A_{k}^{p}\left(t\right)=0$ and therefore, $A_{k}\left(t+1\right)=1$. Alternatively, the evolution of AoI can be written compactly as

(5) $$\begin{array}{c} A_{k}\left(t+1\right)=d_{k}\left(t+1\right)\left(A_{k}^{p}\left(t\right)+1\right)+\left(1-d_{k}\left(t+1\right)\right)\min\left\{A_{k}\left(t\right)+1,M\right\}. \end{array}$$

**Figure 2:**
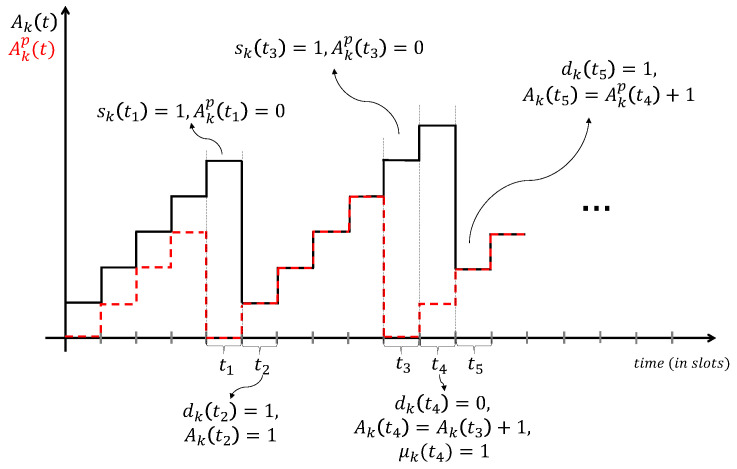
Example of the AoI evolution.

Figure 2 depicts an example of the evolution of $A_{k}^{p}\left(t\right)$ and the AoI at the receiver. At the beginning of the time slot $t_{1}$, user *k* decides to sample and transmit fresh information. Therefore, $A_{k}^{p}\left(t\right)$ becomes zero, and the packet is successfully received by the receiver after one slot. Therefore, in time slot $t_{2}$, the AoI at the receiver is 1. In time slot $t_{3}$, user *k* decides to sample and transmit a fresh packet, but the transmission fails. Thus, we observe by time slot $t_{4}$ the increase on AoI in Figure 2. However, in time slot $t_{4}$, user *k* decides to transmit the old packet which is successfully received by the receiver after one slot.

For each transmission and sampling, we consider a corresponding cost. Let $c_{s}$ and $c_{\mathrm{tr}}$ be the sampling and transmission cost, respectively. We consider that the costs remain the same over time. The cost function for each user *k* at each time slot *t* is described as $c_{k}\left(t\right)=\mu_{k}\left(t\right)c_{tr}+s_{k}\left(t\right)\left(c_{s}+c_{tr}\right),$ and the total system cost in the time slot is described as $c\left(t\right)=\sum_{k=1}^{K}c_{k}\left(t\right).$ The expected average cost and the expected average age for each user are defined as

(6) $$\begin{array}{c} \bar{c}≜\lim_{t\to \infty }\frac{1}{t}\sum_{\tau =0}^{t}E\left\{c\left(\tau \right)\right\},\;{\bar{A}}_{k}≜\lim_{t\to \infty }\frac{1}{t}\sum_{\tau =0}^{t}E\left\{A_{k}\left(\tau \right)\right\},\forall k\in K, \end{array}$$

respectively.

### 2.2. Problem Formulation

With the definitions of AoI and average costs, we define the stochastic optimization problem as following.

(7a) $$\begin{array}{cc} \min_{\mu (t),\;s(t)}\; & \bar{c} \end{array}$$

(7b) $$\begin{array}{cc} s.\;t.\; & {\bar{A}}_{k}\leq A_{k}^{\max},\;\forall k\in K, \end{array}$$

(7c) $$\begin{array}{cc} \; & \sum_{k=1}^{K}\mu_{k}\left(t\right)\leq 1,\;\sum_{k=1}^{K}s_{k}\left(t\right)\leq 1,\;\mu_{k}\left(t\right)+s_{k}\left(t\right)\leq 1,\;\forall k\in K, \end{array}$$

(7d) $$\begin{array}{cc} \; & s\left(t\right),\;\mu \left(t\right)\in {\left\{0,1\right\}}^{K}, \end{array}$$

where $\mu \left(t\right)=[\mu_{1}\left(t\right),\ldots ,\mu_{K}\left(t\right)]$ and $s\left(t\right)=[s_{1}\left(t\right),\ldots ,s_{K}\left(t\right)]$.

Our goal is to find scheduling policies that minimize the total average cost while providing average AoI for each user *k* below the value $A_{k}^{\max}$.

## 3. Scheduling Policies

In this section, we provide three different scheduling policies that satisfy the average AoI constraints and approximately minimize the total average cost. The first policy, named Drift-Plus-Penalty (DPP), is considered a fully centralized policy that takes decisions slot-by-slot in order to minimize the average cost of the system. It is derived by using tools from Lyapunov optimization theory [37]. The second policy, named Old-or-Fresh Randomized Policy (OFRP), is a stationary randomized policy where the scheduler schedules up to one user per time slot with some probability. Then, the scheduled user decides about its action. The third policy, named Fresh-only Randomized Policy (FoRP), is a simple version of the second policy. The scheduler schedules up to one user per time slot with some probability. In this case, the scheduled user has only two options: either to sample and transmit or to remain silent.

Note that for the Lyapunov-based scheduling policy, the scheduler needs complete information about the caches of the users. In addition, an optimization problem is solved slot-by-slot to provide the optimal scheduler’s decision. On the other hand, the stationary randomized policies do not require the scheduler to have any information about the caches of the users; thus, it might be simpler to implement. Based on our analysis, we solve an optimization problem once, in the beginning, for optimizing the scheduling probabilities. These probabilities remain the same over the same, and there is no need for extra computation for every time slot.

### 3.1. Drift-Plus-Penalty Policy

In this section, we provide a dynamic algorithm based on Lyapunov optimization theory that takes decisions slot-by-slot in order to minimize the total average cost while satisfying the time average AoI.

In order to satisfy the average constraints in (7b), we apply the methodology first developed in [37]. In particular, each average constraint in (7b) is mapped into a virtual queue. We show that satisfying the average AoI constraints is equivalent to a queue stability problem.

Let ${\left\{X_{k}\left(t\right)\right\}}_{k\in K}$ be the virtual queues associated with constraints in (7b). The evolution of each queue *k* is shown below

(8) $$\begin{array}{c} X_{k}\left(t+1\right)=\max\left[X_{k}\left(t\right)-A_{k}^{\max},0\right]+A_{k}\left(t+1\right),\;\forall k\in K. \end{array}$$

Process $X_{k}\left(t\right)$ can be viewed as a queue with “arrivals” $A_{k}\left(t\right)$ and service rate $A_{k}^{\max}$.

**Lemma 1.**  *If $X_{k}\left(t\right)$, is rate-stable, ∀k∈K, then the constraints in *(7b)* are satisfied.*

**Proof.**  Using the basic sample property ([37] Lemma 2.1), we have

(9) $$\begin{array}{c} \frac{X_{k}\left(t\right)}{t}-\frac{X_{k}\left(0\right)}{t}\geq \frac{1}{t}\sum_{\tau =0}^{t-1}A_{k}\left(\tau \right)-\frac{1}{t}\sum_{\tau =0}^{t-1}A_{k}^{\max},\;\forall k\in K. \end{array}$$

Therefore, if $X_{k}\left(t\right)$ is rate-stable, so that $\frac{X_{k}\left(t\right)}{t}\to 0$; then, constraints (7b) are satisfied with probability one [38]. □

Before describing the details of the analysis, let us recall a basic theorem [39]. Consider a system with I={1,2,…,I} queues. The number of unfinished jobs of queue *i* is denoted by $q_{i}\left(t\right)$ and $q\left(t\right)={\left\{q_{i}\left(t\right)\right\}}_{i\in I}$. The Lyapunov function and the Lyapunov drift are denoted by L(q(t)) and Δ(q(t))≜E{L(q(t+1))−L(q(t))|q(t)}, respectively.

**Definition 1**  (Lyapunov Function)**.** *A function $L:R^{K}\to R$ is said to be a Lyapunov function if it has the following properties: (1) It is non-decreasing in any of its arguments, (2) $L\left(x\right)\geq 0,\;\forall x\in R^{K}$, (3) L(x)→+∞, as ∥x∥→+∞.*

**Theorem 1**  (Lyapunov Drift)**.** *If there are positive values B, ϵ such that for all time slots t we have $\Delta \left(q\left(t\right)\right)\leq B-\epsilon \sum_{i=1}^{I}q_{i}\left(t\right)$, then the system q(t) is strongly stable [39].*

The intuition behind Theorem 1 is that if we have a queueing system and we provide a policy for which the Lyapunov drift is bounded by a constant value B>0 and the sum of the length of the queues multiplied by a negative value for every time slot, then the system can be stabilized.

The DPP algorithm is designed to minimize the sum of the Lyapunov drift and a penalty function ([37] Chapter 3). First, we define the Lyapunov drift as

(10) $$\begin{array}{c} \Delta \left(X\left(t\right)\right)=E\{L\left(X\left(t+1\right)\right)-L\left(X\left(t\right)\right)|S_{t}\}, \end{array}$$

where $S_{t}={\left\{A_{k}\left(t\right),X_{k}\left(t\right)\right\}}_{k\in K}$ is the network state at slot *t*, and $X\left(t\right)={\left\{X_{k}\left(t\right)\right\}}_{k\in K}$. The associated Lyapunov function is defined as $L\left(X\left(t\right)\right)=\frac{1}{2}\sum_{k=1}^{K}X_{k}^{2}\left(t\right).$ The above expectations are with respect to the channel randomness and the scheduling policy. We apply the DPP algorithm in order to minimize the total average cost (penalty function) while stabilizing the virtual queues, i.e., providing the average AoI below the given value for each user. In particular, this approach seeks to minimize an upper bound of the following expression

(11) $$\begin{array}{c} \Delta \left(X\left(t\right)\right)+VE\left\{c\left(t\right)|S_{t}\right\}, \end{array}$$

where *V* is an importance weight factor. An upper bound for the expression in (11) is shown below

(12) $$\begin{array}{cc} \Delta \left(X\left(t\right)\right)+VE\left\{c\left(t\right)|S_{t}\right\}\; & \leq B+\sum_{k=1}^{K}X_{k}\left(t\right)E\{W_{k}\left(t\right)\left(A_{k}^{p}\left(t\right)+1\right)+\left(1-W_{k}\left(t\right)\right)\times \\ \; & \min\left[A_{k}\left(t\right)+1,M\right]\left|S_{t}\right\}-A_{k}^{\max}+VE\left\{c\left(t\right)|S_{t}\right\}, \end{array}$$

where $B\geq \sum_{k=1}^{K}\frac{E\left\{A_{k}^{2}\left(t+1\right)|S_{t}\right\}+{\left(A_{k}^{\max}\right)}^{2}}{2}$, and $W_{k}\left(t\right)=p_{k}s_{k}\left(t\right)+p_{k}\mu_{k}\left(t\right)$. The complete derivation of the above bound can be found in Appendix A. If we set $B=\sum_{k=1}^{K}\frac{M^{2}+{\left(A_{k}^{\max}\right)}^{2}}{2}\geq \sum_{k=1}^{K}\frac{E\left\{A_{k}^{2}\left(t+1\right)|S_{t}\right\}+{\left(A_{k}^{\max}\right)}^{2}}{2}$, we see that *B* is a constant and it does not depend on the scheduling decisions over slots. Therefore, we can exclude *B* from the optimization problem. The DPP algorithm takes sampling and transmission decisions at each time slot by solving the following optimization problem.

(13a) $$\begin{array}{cc} \min_{\mu (t),\;s(t)}\; & \sum_{k=1}^{K}\{X_{k}\left(t\right)[\left(A_{k}^{p}\left(t\right)+1\right)W_{k}\left(t\right)+\\ \; & \min\left\{\left(A_{k}\left(t\right)+1\right),M\right\}\left(1-W_{k}\left(t\right)\right)-A_{k}^{\max}]\}\\ \; & +Vc(t) \end{array}$$

(13b) $$\begin{array}{cc} s.\;t.\; & \sum_{k=1}^{K}\mu_{k}\left(t\right)\leq 1,\;\sum_{k=1}^{K}s_{k}\left(t\right)\leq 1,\mu_{k}\left(t\right)+s_{k}\left(t\right)\leq 1,\;\forall k\in K, \end{array}$$

(13c) $$\begin{array}{cc} \; & s\left(t\right),\;\mu \left(t\right)\in {\left\{0,1\right\}}^{K}. \end{array}$$

Also, we define function $y_{k}\left(t+1\right)$, ∀k as following

$$\begin{array}{cc} \; & y_{k}\left(t+1\right)=A_{k}\left(t+1\right)-A_{k}^{\max}\\ \; & =\left(A_{k}^{p}\left(t\right)+1\right)\left(p_{k}s_{k}\left(t\right)+p_{k}\mu_{k}\left(t\right)\right)+\min\left\{\left(A_{k}\left(t\right)+1\right),M\right\}(1-p_{k}s_{k}\left(t\right)-\mu_{k}\left(t\right)p_{k}-A_{k}^{\max}. \end{array}$$

**Lemma 2.**  *We consider a class of stationary possible randomized policies denoted by *Ω*. A policy* ω(t) *that belongs to the class* Ω* is an i.i.d. process that takes probabilistic decisions independently of the states of the caches at every time slot t. Let* $y_{k}\left(t\right)=A_{k}\left(t\right)-A_{k}^{max}$ *and* c(t) *be the total system cost. Then, if the problem in *(7)* is strictly feasible, and the second moments of* $y_{k}\left(t\right)$ *and* c(t) *are bounded, then for any* ϵ>0, *there is an* ω(t) *policy under which the following holds:*

(14) $$\begin{array}{c} E\left\{y_{k}^{*}\left(t+1\right)\right\}\leq \epsilon ,\;E\left\{c^{*}\left(t\right)\right\}=c_{\omega }\left(\epsilon \right)\leq c^{\mathrm{opt}}+\epsilon , \end{array}$$

*where* $y_{k}^{*}\left(t+1\right)$ *and* $c^{*}\left(t\right)$ *are the resulting values of the ω policy,* $c^{\mathrm{opt}}$ *is best objective function in *(7)* achievable by any stationary randomized policy, and* $c_{\omega }\left(\epsilon \right)$ *is a feasible suboptimal solution to the problem in *(7)* that can be achieved by an ω stationary randomized policy.*

**Proof.**  We consider the cases in which the problem in (7) is strictly feasible. Furthermore, by definition, $A_{k}\left(t\right)$, ∀k is bounded by finite value *M*; therefore, the first and the second moments of $A_{k}\left(t\right)$, are also bounded. Furthermore, the cost function c(t) is also bounded by the sum of constant values $c_{s}+c_{\mathrm{tr}}$, i.e., the sampling and the transmission cost, respectively. Therefore, we have $1\leq E\left\{A_{k}^{2}\left(t\right)\right\}\leq M^{2},\;0\leq E\left\{c^{2}\left(t\right)\right\}\leq {\left(c_{\mathrm{tr}}+c_{s}\right)}^{2}K^{2}.$ Then, the boundness assumptions in ([37] Ch. 4.2.1) are satisfied. Therefore, from Theorem 4.5 in [37], we obtain the result. □

**Theorem 2.**  *The DPP algorithm satisfies any feasible set of the maximum AoI constraints.*

**Proof.**  See Appendix B. □

**Theorem 3**  (Optimality of the DPP algorithm)**.** *After applying the DPP algorithm, we obtain an expected average cost that is bounded as*

(15) $$\begin{array}{c} \lim_{t\to \infty }\sup\frac{1}{t}\sum_{\tau =0}^{t-1}E\left\{c\left(\tau \right)\right\}\leq c^{\mathrm{opt}}+\frac{B}{V}. \end{array}$$

**Proof.**  See Appendix C. □

**Remark 1.**  *Theorem 2 indicates that the DPP algorithm provides an arbitrarily close solution to the optimal. We can obtain better performance in terms of average cost by increasing the value of V. However, by increasing the value of V, the values of the length of the virtual queues will increase as well. Therefore, there is a trade-off between the average cost and average age.*

**Remark 2.**  *The drift-plus-penalty algorithm is based on the Lyapunov optimization theory. The Lyapunov drift is used to guarantee the stability of the virtual queues, and therefore, the satisfaction of the time average constraints. The Lyapunov drift is the expected change in the values of* X(t) *from one slot to another. By providing an upper bound on the Lyapunov drift *(11)*, we guarantee that by minimizing this bound that is finite, the length of the virtual queues is also bounded and therefore stable. The drift-plus-penalty seeks to minimize the bound on the Lyapunov drift and the total system cost.*

We prove that, if we solve the weighted optimization problem that minimizes the bound of the Lyapunov drift plus the penalty, we achieve the minimum cost asymptotically and we satisfy the constraints. The weighted optimization problem consists of two kinds of weights. The first is the importance factor *V* multiplied by the total system cost. The second kind of weight is the values of the virtual queues. Therefore, when the length of a virtual queue is quite large and larger than the corresponding cost, we allocate resources to the corresponding user in order to reduce the AoI at the receiver. On the other hand, if the length of a virtual queue is low, then implicitly we know that the corresponding user has been allocated a sufficient amount of resources so far such that its AoI at the receiver is low.

### 3.2. First Stationary Randomized Policy—Fresh or Old Randomized Policy

We consider a scheduler that probabilistically decides which user will be scheduled at every time slot. The scheduler schedules up to one user per time slot. The probability of the scheduler allocating user *k* is $\alpha_{k}$. Therefore, $\sum_{k=1}^{K}\alpha_{k}=1$. If a user *k* is scheduled in a slot, then it decides by itself the following actions with the corresponding probabilities:

- If there is a packet in the cache, user *k* (1) samples and transmits with probability (w.p.) $u_{k}$, (2) transmits an old packet w.p. $q_{k}$, (3) remains silent w.p. $1-u_{k}-q_{k}$.
- If there is no packet in the cache, user *k* (1) samples and transmits w.p. $u_{k}^{\prime }$, (2) remains silent w.p. $1-u_{k}^{\prime }$.

Note that $\mathrm{Pr}\left\{s_{k}\left(t\right)=1|Q_{k}\left(t\right)=1\right\}=\alpha_{k}u_{k},\mathrm{Pr}\left\{s_{k}\left(t\right)=1|Q_{k}\left(t\right)=0\right\}=\alpha_{k}u_{k}^{\prime }.$ Furthermore, $\mathrm{Pr}\left\{\mu_{k}\left(t\right)=1|Q_{k}\left(t\right)=1\right\}=\alpha_{k}q_{k}.$ In the case where $A_{k}\left(t\right)=A_{k}^{p}\left(t\right)+1$, there is no advantage regarding the AoI to transmit an old packet because after one slot the AoI of the received packet will be $A_{k}\left(t+1\right)=A_{k}^{p}\left(t\right)+1=A_{k}\left(t\right)$. Therefore, in this case, we discard the packet in the cache, and then the options are either to sample and transmit a fresh packet or to remain silent.

In order to select a set of decision probabilities that satisfies the constraints while minimizing the average cost, we need to derive the average cost and the average AoI as a function of those probabilities. Here, we focus on the case of a single user. However, the same methodology is applied for each user independently in a multiple-user scenario. For the sake of the presentation, we omit the subscript *k* without sacrificing clarity. In order to obtain the expression for the distribution of the AoI and the average AoI, we model the evolution of the AoI at the receiver, $A_{t}=A\left(t\right)$, and the evolution of the waiting time in the cache, $A_{t}^{p}=A^{p}\left(t-1\right)+1$, as one two dimensional DTMC. The transition to the next state depends on the current state, i.e., $\left(A_{t}^{p},A_{t}\right)$, on the decision of the scheduler, and on the decision of the scheduled user. The DTMC $\left\{\left(A_{t}^{p},A_{t}\right)\right\}$ is described by the following transition probability, $\forall i,j,m,l,P_{\left(i,j)\to (m,l\right)}=\mathrm{Pr}\left\{A_{t+1}^{p}=m,A_{t+1}=l\;|\;A_{t}^{p}=i,A_{t}=j\right\}.$ Note that we do not need to include the state of the cache in the Markov chain because the values of $A_{t}^{p}$ and $A_{t}$ can indicate to us whether the cache has a packet or not. For example, if $A_{t}^{p}=A_{t}$, we know that at the beginning of time slot *t*, we have a successful transmission. Therefore, the cache of the user is empty after the successful transmission and the only options are either to sample and transmit or remain idle. The transition to each state depends on the events that happened in the previous slot. The events are (i) the decision of the scheduler, (ii) the decision of the scheduled user, and, if there is any, (iii) the outcome of the transmission. We categorize the states according to the values of $A_{t}^{p}$ and $A_{t}$ below.

If i≤j−1, j<M, i<M−1: In this case, there is a packet in the cache sampled during the previous slots, i.e., Q(t)=1. The transition probabilities are:
  (16) $$\begin{array}{c} P_{\left(i,j)\to (m,l\right)}=\left\{\begin{array}{c} \alpha up\;\mathrm{if},\;m=1\;\mathrm{and}\;l=1,\;\\ \alpha u(1-p),\;\mathrm{if}\;m=1\;\mathrm{and}\;l=j+1,\;\\ \alpha qp,\;\mathrm{if}\;m=i+1\;\mathrm{and}\;l=i+1,\;\\ 1-\alpha u-qp\alpha ,\;\mathrm{if}\;m=i+1,\;l=j+1. \end{array}\right. \end{array}$$
If j<M, i<M−1: In this case, there is no packet in the cache, i.e., Q(t)=0. The transition probabilities are described below:
  (17) $$\begin{array}{c} P_{\left(i,j)\to (m,l\right)}=\left\{\begin{array}{c} \alpha u^{\prime }p,\;\mathrm{if}\;m=1,\;l=1,\\ \alpha u^{\prime }\left(1-p\right),\;\mathrm{if}\;m=1,\;l=j+1,\\ 1-\alpha u^{\prime },\;\mathrm{if}\;m=i+1,\;l=j+1. \end{array}\right. \end{array}$$
If j=M, i<M−1: In this case, we have a packet in the cache.
  (18) $$\begin{array}{c} P_{\left(i,j)\to (m,l\right)}=\left\{\begin{array}{c} \alpha up,\;\mathrm{if}\;m=1,\;l=1,\\ \alpha u(1-p),\;\mathrm{if}\;m=1,\;l=M,\\ \alpha qp,\;\mathrm{if}\;m=i+1,\;l=i+1,\\ 1-\alpha u-qp\alpha ,\;\mathrm{if}\;m=i+1,\;l=M. \end{array}\right. \end{array}$$
If i=1, j=M. In this case, there is an old packet packet in the cache, Q(t)=1. The transition probabilities are described below:
  (19) $$\begin{array}{c} P_{\left(i,j)\to (m,l\right)}=\left\{\begin{array}{c} \alpha up,\;\mathrm{if}\;m=1\;\mathrm{and}\;l=1,\\ \alpha u(1-p),\;\mathrm{if}\;m=1\;\mathrm{and}\;l=M,\\ \alpha qp,\;\mathrm{if}\;m=2\;\mathrm{and}\;l=2,\\ 1-\alpha u-\alpha qp\;\mathrm{if}\;m=i+1\;\mathrm{and}\;l=M. \end{array}\right. \end{array}$$
AoI in the boundaries: i=M−1, j=M: In this case, even if the cache has an old packet the user drops this packet because a successful transmission of the old packet will not improve the AoI at the receiver. The transition probabilities are described below:
  (20) $$\begin{array}{c} P_{\left(i,j)\to (m,l\right)}=\left\{\begin{array}{c} 1-\alpha u^{\prime },\;\mathrm{if}\;m=M-1,\;l=m,\\ \alpha u^{\prime }p,\;\mathrm{if}\;m=1,\;l=1,\\ \alpha u^{\prime }\left(1-p\right),\;\mathrm{if}\;m=1,\;l=M. \end{array}\right. \end{array}$$

We have now completely described the transition matrix, P, and we can obtain the steady-state distribution of AoI at the receiver. We consider one Markov Chain for each user *k* and its transition matrix is denoted by $P_{\left(k\right)}$.

We denote the steady-state distribution of the AoI by a row vector

(21) $$\begin{array}{c} \pi_{\left(k\right)}=\left[\pi_{0,1}^{\left(k\right)},\pi_{0,2}^{\left(k\right)},\ldots ,\pi_{0,M}^{\left(k\right)},\pi_{1,2}^{\left(k\right)},\ldots \pi_{1,M}^{\left(k\right)},\ldots \right]. \end{array}$$

We obtain $\pi_{\left(k\right)}$ by solving the following linear system of equations $\begin{array}{c} \pi_{\left(k\right)}P_{\left(k\right)}=\pi_{\left(k\right)},\;\pi_{\left(k\right)}1=1, \end{array}$ where 1 is a column vector with all its elements being one. We can obtain the steady-state distribution by applying numerical methods, e.g., EigenValue-Decomposition (EVD). The average AoI for each user *k* is calculated as ${\bar{A}}_{k}=\sum_{i=0}^{M-1}\sum_{j=i+1}^{M}j\pi_{i,j}^{\left(k\right)}.$ We set $\theta =\sum_{i=1}^{M-1}\sum_{j=i}^{i+1}\pi_{i,j}^{\left(k\right)}$, where θ is the probability of the cache being empty. The average cost for each user *k* is calculated as

(22) $$\begin{array}{c} {\bar{c}}_{k}=\theta \alpha_{k}u_{k}^{\prime }\left(c_{tr}+c_{s}\right)+\left(1-\theta \right)\left(\alpha_{k}\left(q_{k}c_{tr}+u_{k}\left(c_{tr}+c_{s}\right)\right)\right). \end{array}$$

In order to optimize the parameters $\alpha_{k}$, $u_{k}^{\prime }$, $q_{k}$, we select a step for selecting combinations of the possible values of the probabilities. We discretize the space of the probabilities and we take the corresponding combinations. The smallest the step the highest the number of combinations and, therefore, the complexity. We find the combinations for which the constraints are satisfied, and we select the combination of the probabilities that provide the lowest total system cost.

### 3.3. Fresh-Only Randomized Policy

Here, we propose a simple randomized policy for which we obtain closed-form expressions. At each time slot, the scheduler probabilistically schedules up to one user at each time slot. Then, the scheduled user decides either to sample and transmit or to remain idle (no option for transmission of an old packet). In particular, the scheduler schedules user *k* with probability $\alpha_{k}^{\prime }$ where $\sum_{k=1}^{K}\alpha_{k}^{\prime }=1$. If user *k* is scheduled, then (1) it decides to sample and transmit a fresh packet with probability $\phi_{k}$ and (2) it decides to remain silent with $1-\phi_{k}$. In order to obtain the expression for the average AoI for each user *k*, and the total average cost we model the system as one DTMC. In the following analysis, we omit subscript *k* without sacrificing clarity. The same methodology is applied for each user *k*. We model the AoI for each user *k* as one Markov chain, where $\delta =\alpha^{\prime }\phi p$. The transition matrix, $P^{\prime }$, of the Markov chain is shown below

$$\begin{array}{cc} P^{\prime } & =\left[\begin{array}{cccccc} \delta & 1-\delta & 0 & 0 & \cdots & 0\\ \delta & 0 & 1-\delta & 0 & \cdots & 0\\ \vdots & \vdots & & \ddots \\ \delta & 0 & 0 & 0 & \cdots & 1-\delta \\ \delta & 0 & 0 & 0 & \cdots & 1-\delta \end{array}\right]. \end{array}$$

We consider one Markov chain for each user *k*. The transition matrix of each user *k* is denoted by $P_{\left(k\right)}^{\prime }$. We denote the steady-state distribution of the AoI by a row vector as $\pi_{\left(k\right)}^{\prime }=\left[\pi_{1}^{\left(k\right)},\pi_{2}^{\left(k\right)},\ldots ,\pi_{M}^{\left(k\right)}\right].$ In order to obtain the steady-state distribution of the AoI, we solve the following linear system of equations $\pi_{\left(k\right)}^{\prime }P_{\left(k\right)}^{\prime }=\pi_{\left(k\right)}^{\prime },\;\pi_{\left(k\right)}^{\prime }1=1.$ After some calculations, we obtain the steady-state distribution of the AoI at the receiver, as follows: $\pi_{i}^{\left(k\right)}=\delta_{k}{\left(1-\delta_{k}\right)}^{i-1},\;\mathrm{for}\;i<M\pi_{M}^{\left(k\right)}={\left(1-\delta_{k}\right)}^{M-1},\;\mathrm{for}\;i=M.$ We set ${\bar{\delta }}_{k}=1-\delta_{k}$, and the average AoI for each user *k* is calculated as

(23) $$\begin{array}{cc} {\bar{A}}_{k} & =\sum_{i=1}^{M}i\pi_{i}^{\left(k\right)}=\sum_{i=1}^{M-1}i\delta_{k}{\bar{\delta }}_{k}^{i-1}+M{\left({\bar{\delta }}_{k}\right)}^{M-1}=\frac{\delta_{k}}{{\bar{\delta }}_{k}}\sum_{i=1}^{M-1}i{\bar{\delta }}_{k}^{i}+M{\left({\bar{\delta }}_{k}\right)}^{M-1}\\ \; & \overset{\left(a\right)}{=}\frac{\left(M-1\right){\bar{\delta_{k}}}^{M}-M{\bar{\delta_{k}}}^{M-1}+1}{\delta_{k}}+M{\bar{\delta_{k}}}^{M-1}, \end{array}$$

where (a) follows by applying $\sum_{i=0}^{n}ic^{i}=\frac{nc^{n+2}-\left(n+1\right)c^{n+1}+c}{{\left(c-1\right)}^{2}},\;c\neq 1$. The average cost for each user *k* is ${\bar{c}}_{k}=\left(c_{tr}+c_{s}\right)\left(\alpha_{k}^{\prime }\phi_{k}\right).$

In order to minimize the probabilities, we calculate the average AoI for every combination of the value of $\phi_{k}$ and $\alpha_{k}^{\prime }$ between 0 and 1 with a step Δ, and if the constraint is satisfied, we calculate the corresponding cost. The decision probabilities for every user are the ones for which the average AoI constraints are satisfied and the cost is minimized.

## 4. Simulation and Numerical Results

In this section, we provide results that show the performance of the proposed policies regarding the total average cost, for various scenarios. We compare the scheduling policies for different success probabilities as well as different AoI constraints. Furthermore, we analyze the scheduling decisions for the DPP and OFRP policy for different values of costs, i.e., when the sampling cost is larger than the transmission cost.

In order to find the optimal probabilities of the randomized policies, we apply an exhaustive search. In particular, we take values of the probabilities that are between 0 and 1, with a step that equals 0.01. We set these values in the derived expressions and we find the combinations for which the AoI constraints are satisfied and the total average cost is minimized. For the DPP policy, each simulation has run for $10^{6}$ slots, and the importance factor, *V*, is equal to 800 for each case. We perform our simulations in MATLAB (https://www.mathworks.com/products/matlab.html, accessed on 11 October 2024).

### 4.1. Performance of the Scheduling Policies

**Figure 3:**
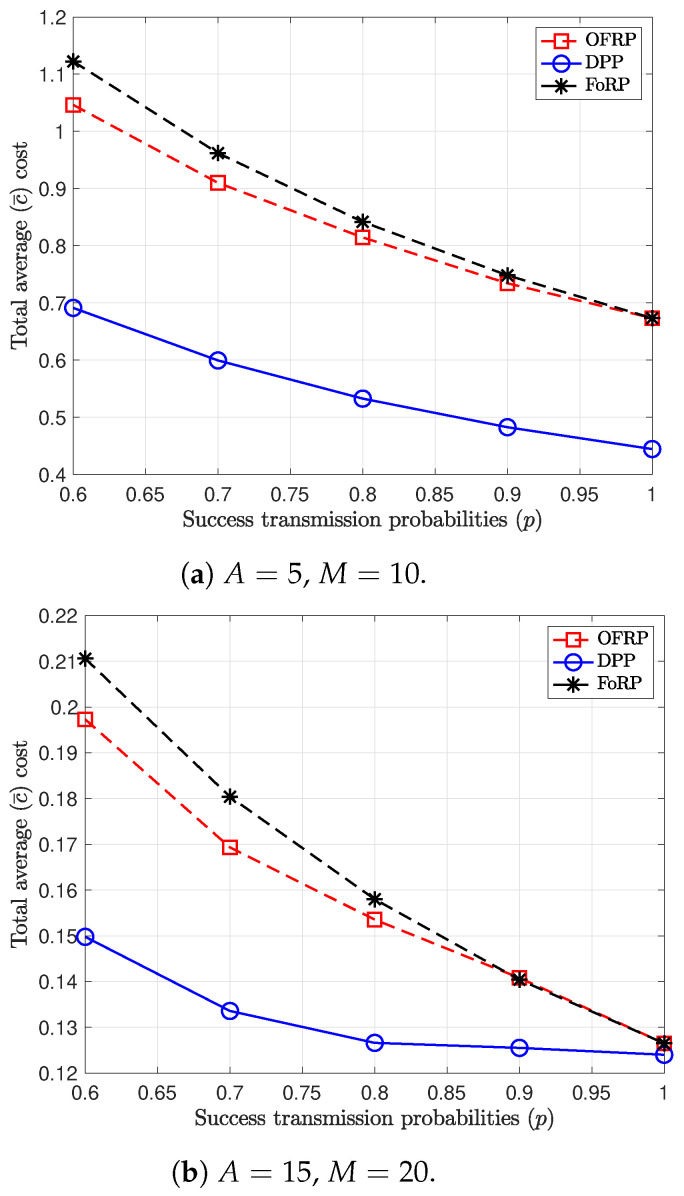
Performance comparison between the proposed policies for different success probabilities.

In Figure 3, we provide results for different values of the success transmission probabilities and different values of the AoI constraints. In particular, Figure 3a depicts the total average cost for each scheduling policy, where the values of the AoI constraints are A=5, and the AoI threshold, *M*, is equal to 10. We observe that when the success probabilities approach 1, the performance of OFRP and FoRP tend to be identical, and for p=1, the performance of the randomized policies is equal. The reason is that for high success probabilities, the probability of the cache having a packet is quite small, and for error-free channels, the caches of the users are always empty. Therefore, OFRP always schedules the users to sample and transmit or to remain silent. In this case, the two randomized policies have identical behavior.

On the other hand, when the success transmission probabilities are small and A=15, the difference between the randomized policies increases regarding the performance, as shown in Figure 3b. In this case, the scheduling policies utilize the flexibility that is given by the large value of $A_{1}^{\max}$. Therefore, in this case, the option of transmitting an old packet reduces the total average cost.

**Figure 4:**
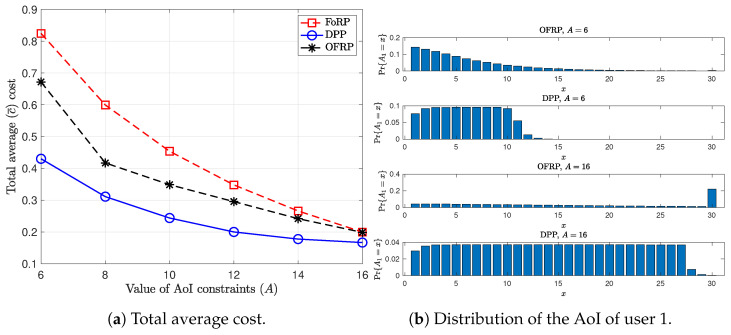
Performance comparison between the proposed policies for values of the AoI constraints. p=0.8, M=25.

In Figure 4a, we provide results that show the performance of the scheduling policies as the values of the AoI constraints, i.e., *A*, increase. We consider that the success transmission probabilities are equal to 0.8, and the AoI threshold, *M*, is 25. Obviously, for larger values of the AoI constraints, the total average cost decreases for all the scheduling policies. Furthermore, we observe that as the hardness of the AoI constraints decreases, the difference in the performance between OFRP and DPP decreases as well. In this case, the OFRP utilizes that the AoI cannot be larger a value and, therefore, it gains in cost. On the other hand, we observe that the DPP policy allows the value of AoI to reach the threshold only for a small percentage of time (see the distribution of the AoI in Figure 4b, for $A_{1}^{\max}=16$). However, the DPP algorithm still outperforms the randomized policies regarding the total average cost.

**Figure 5:**
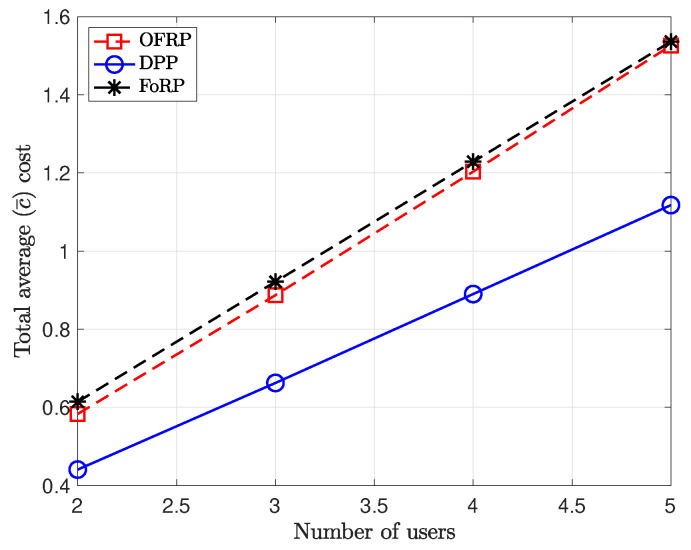
A multi-user scenario. M=15, value of AoI constraints: $A_{1}^{\max}=A_{2}^{\max}=7$. Success transmission probabilities: $p_{1}=p_{2}=0.8$.

**Figure 6:**
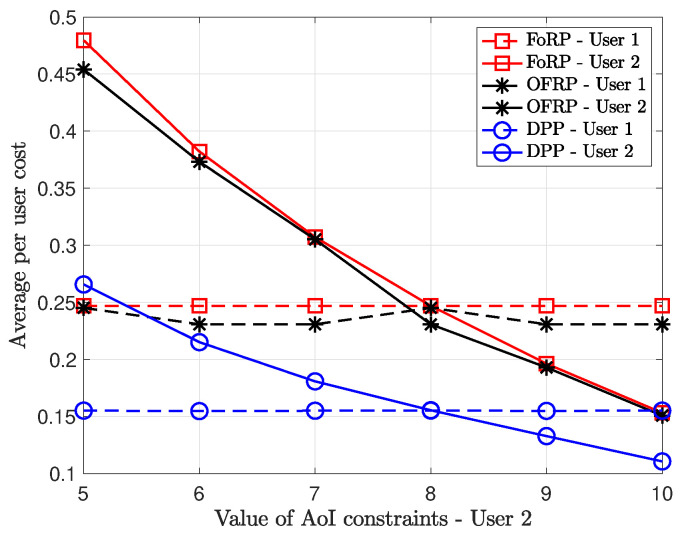
$p_{1}=p_{2}=0.8$. M=20. $A_{1}^{\max}=8$.

In addition, we provide results for multi-user scenarios in Figure 5. The success transmission probabilities are equal to 0.8, and the values of AoI constraints are equal to 7. We observe that the total average cost increases linearly with respect to the number of users in the system. However, the total average cost achieved by applying stationary randomized policies increases faster than the one achieved by the DPP algorithm. Therefore, the DPP algorithm can be considered more robust in terms of the total average with respect to the number of users in the system. Furthermore, in Figure 6, we provide results for two non-symmetric users. In particular, the value of the AoI constraint of user 1 is equal to 8. We provide results for the average per-user cost as the value of AoI constraint of user 2 increases. We observe that as the value of AoI increases, the average per-user cost decreases almost linearly for user 2. On the other hand, the cost for user 1 remains almost constant since the value of AoI does not change.

### 4.2. Scheduling Decisions and Performance Comparison

**Figure 7:**
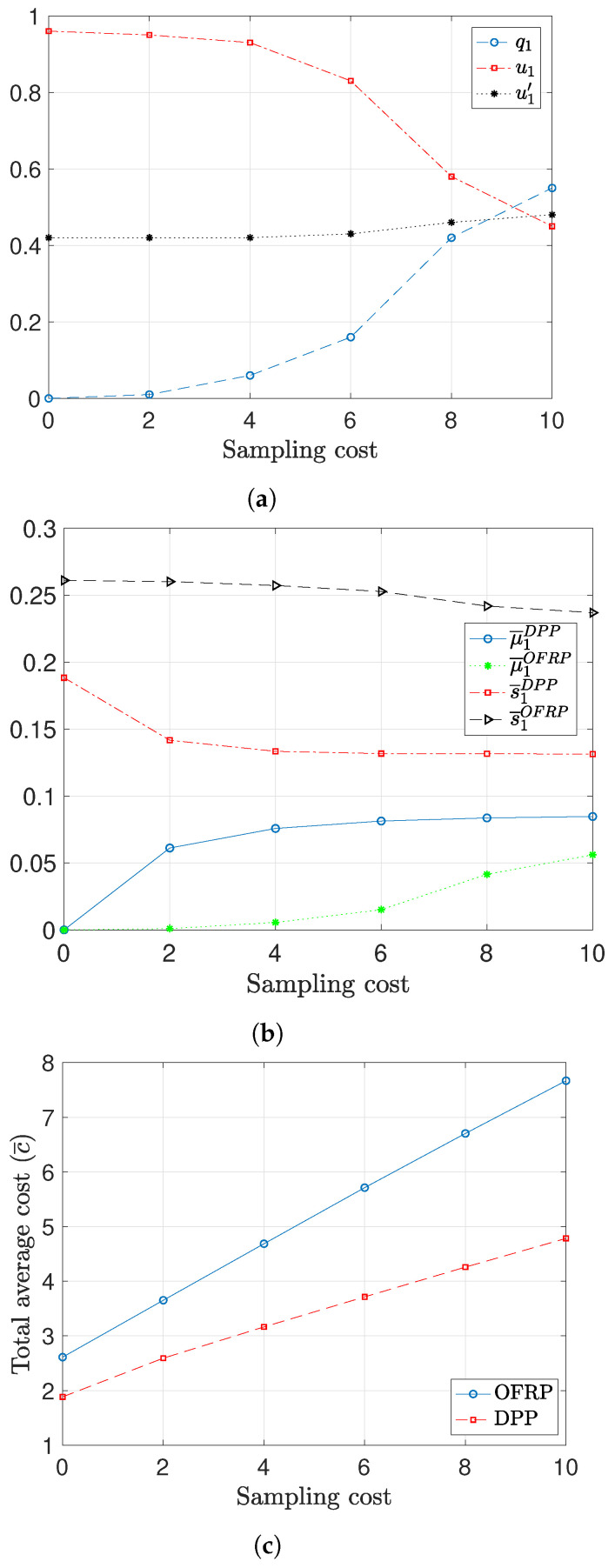
Scheduling decisions and performance comparison for different values of the sampling cost, $c_{\mathrm{tr}}=5$, A=5, M=10. (**a**) Optimized probabilities of the OFRP policy. (**b**) Average values of $\mu_{1}$ and $s_{1}$. (**c**) Performance comparison.

**Figure 8:**
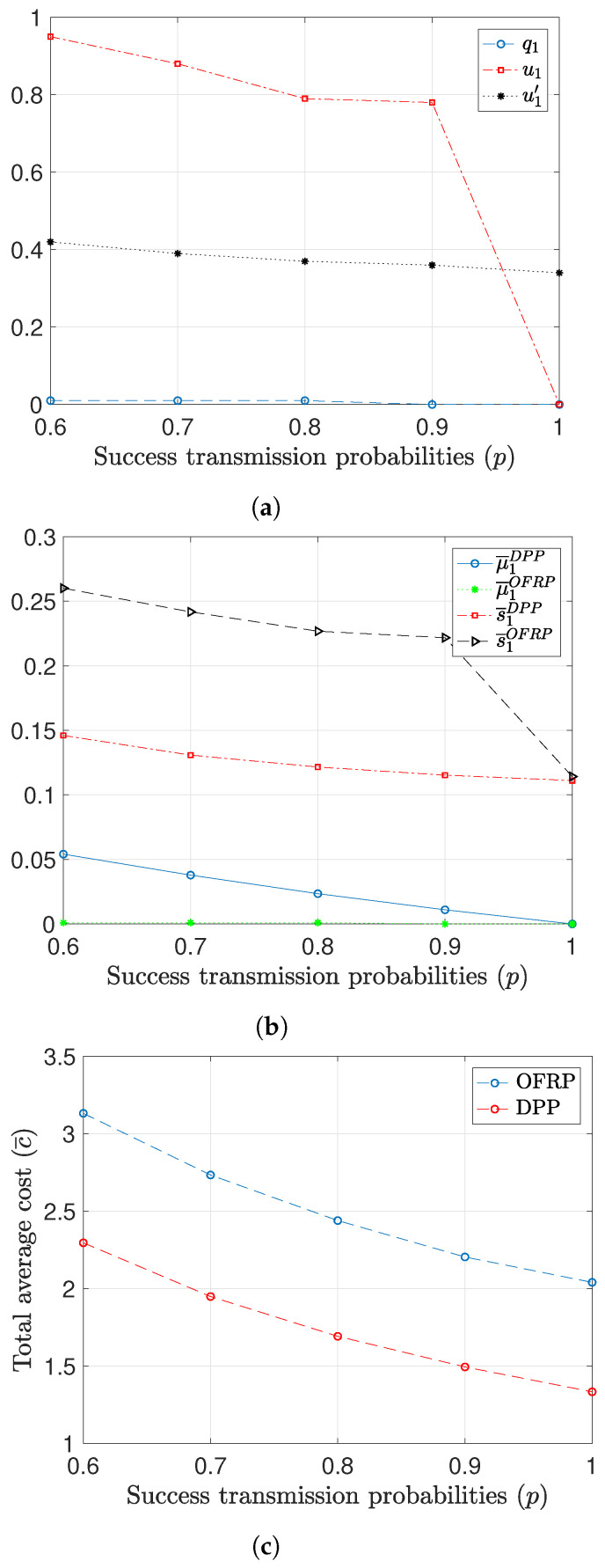
Scheduling decisions for different values of the success probabilities. A=5, M=10, $c_{\mathrm{tr}}=5$. (**a**) Optimized probabilities of the OFRP policy. (**b**) Average values of $\mu_{1}$ and $s_{1}$. (**c**) Total average cost.

In Figure 7 and Figure 8, we provide results that show the scheduling decisions of the scheduling policies for different values of the sampling and transmission cost. We consider a system with two symmetric users. More specifically, we consider that the users have the same success transmission probabilities, the same value of AoI constraints and the same transmission and sampling costs. We observe that in this case, the optimized scheduling probabilities are $\alpha_{1}=\alpha_{2}=0.5$. In these plots, we focus on the first user (user 1) to show the behavior of the policies for different transmission policies. In the figures with total cost, we depict the total average cost for both users. In Figure 7, we consider that the transmission cost is $c_{\mathrm{tr}}=5$, and the AoI constraints A=5, where M=10. Figure 7a depicts the optimized probabilities of the OFRP policy for different values of the sampling cost. We observe that as the sampling cost takes values larger than the transmission cost, $u_{1}$ decreases and $q_{1}$ increases. Note that these probabilities are conditional probabilities. In particular, $q_{1}$ is the probability of scheduling user 1 to transmit an old packet given that there is an old packet in the cache, and $u_{1}$ is the probability of scheduling user 1 to transmit a fresh packet given that there is an old packet in the cache.

In Figure 7b, we compare the values of ${\bar{\mu }}_{1}$ and ${\bar{s}}_{1}$ of the DPP policy with those of the OFRP policy. We observe that ${\bar{s}}_{1}^{\mathrm{DPP}}<{\bar{s}}_{1}^{\mathrm{OFRP}}$ and ${\bar{\mu }}_{1}^{\mathrm{DPP}}>{\bar{\mu }}_{1}^{\mathrm{OFRP}}$. Therefore, with DPP, the user transmits old packets more frequently than OFRP. Thus, the DPP utilizes more efficiently the option of transmitting an old packet than OFRP, and as a result, the corresponding cost is smaller for the case of the DPP policy, as shown in Figure 7c.

In Figure 8, we provide results that show the behavior of the DPP and OFRP policies regarding the scheduling decisions for different values of the success probabilities. The values of the AoI are A=5, and the transmission and sampling costs are $c_{\mathrm{tr}}=5$ and $c_{s}=1$, respectively. Figure 8 depicts the scheduling probabilities for the OFRP policy. Although the sampling cost is significantly smaller than the transmission cost, we observe that for small values of *p*, the OFRP utilizes the option of transmitting an old packet. The reason is that the sampling cost is small but not negligible. We also see that the DPP policy utilizes option of transmitting an old packet more efficiently the than the OFRP, as shown in Figure 8b. As a result, the performance of the DPP policy is better than that of the OFRP policy, as shown in Figure 8c.

### 4.3. Performance of DPP for Different Values of V

**Figure 9:**
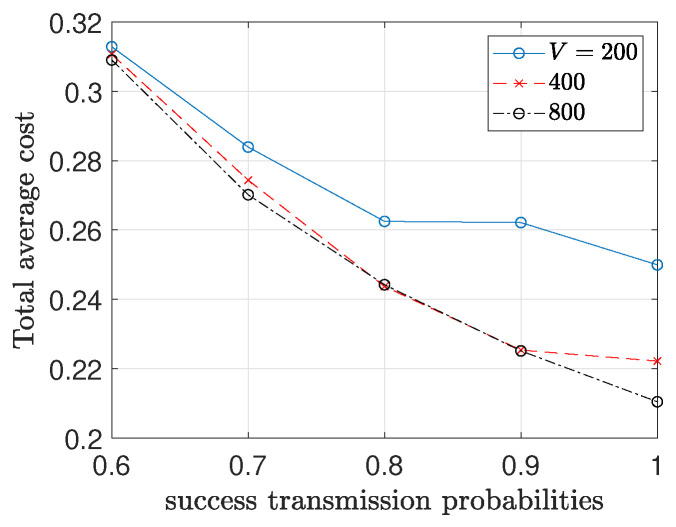
AoI values for different V.

**Figure 10:**
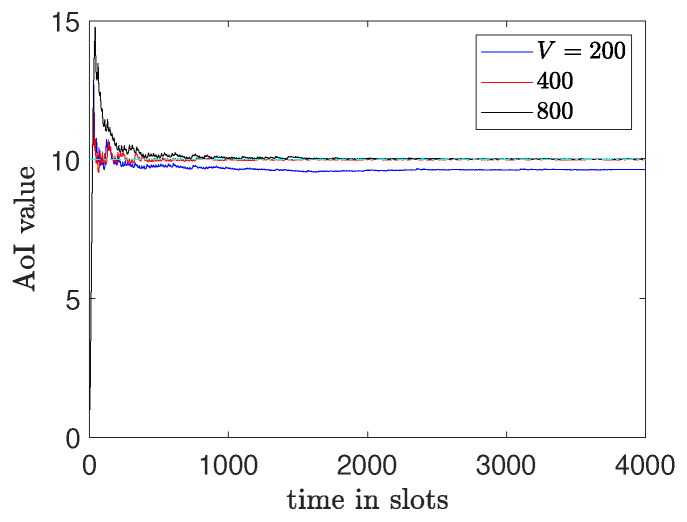
AoI values for different V.

In Figure 9, we observe the total average cost changes for different values of *V*. It is shown that as the value of *V* increases the total average cost decreases. However, there is an impact on the convergence speed for the AoI constraints as it shown in Figure 10.

## 5. Conclusions

In this work, we consider the total cost minimization problem while guaranteeing the required freshness of the data at the receiver. We propose three scheduling policies: one dynamic policy that optimizes the decisions slot-by-slot, and two stationary randomized policies of which the probabilities remain stable during the time. Simulation results show the importance of having the option to transmit an old packet instead of sampling a fresh one each time that a user is scheduled. In particular, for the cases in which the sampling cost is larger than the transmission cost, we observe that by transmitting an old packet, we can significantly improve the total average cost while satisfying the average AoI constraints.

## Data Availability

No new data were created or analyzed in this study. Data sharing is not applicable to this article.

## Appendix A. Upper Bound on the Drift

By using the fact that ${\left(\max[Q-b,0]+A\right)}^{2}\leq Q^{2}+A^{2}+b^{2}+2Q\left(A-b\right)$, we can rewrite (8) as $X_{k}^{2}\left(t+1\right)\leq X_{k}^{2}\left(t\right)+A_{k}^{2}\left(t+1\right)+{\left(A_{k}^{\max}\right)}^{2}+2X_{k}\left(t\right)\left(A_{k}\left(t+1\right)-\left(A_{k}^{\max}\right)\right).$ By rearranging the terms and dividing by 2, we obtain

(A1) $$\begin{array}{c} \sum_{k=1}^{K}X_{k}^{2}\left(t+1\right)-X_{k}^{2}\left(t\right)\leq \sum_{k=1}^{K}\frac{A_{k}^{2}\left(t+1\right)+{\left(A_{k}^{\max}\right)}^{2}+2X_{k}\left(t\right)\left(A_{k}\left(t+1\right)-A_{k}^{\max}\right)}{2}. \end{array}$$

By taking the conditional expectation in (A1), we obtain

(A2) $$\begin{array}{c} \Delta \left(X\left(t\right)\right)\leq \sum_{k=1}^{K}\frac{E\left\{A_{k}^{2}\left(t+1\right)|S_{t}\right\}+{\left(A_{k}^{\max}\right)}^{2}}{2}+\sum_{k=1}^{K}X_{k}\left(t\right)\left[E\left\{A_{k}\left(t+1\right)|S_{t}\right\}-A_{k}^{\max}\right]. \end{array}$$

In order to proceed, we have to calculate $E\{A_{k}\left(t+1\right)|S_{t}\}$. By utilizing the expression for the evolution of AoI in (5), we obtain

(A3) $$\begin{array}{cc} \; & E\left\{A_{k}\left(t+1\right)|S_{t}\right\}=E\left\{\left(A_{k}^{p}\left(t\right)+1\right)d_{k}\left(t+1\right)+\left(1-d_{k}\left(t+1\right)\right)\min\left\{A_{k}\left(t\right)+1,M\right\}|S_{t}\right\}\\ \; & =E\left\{p_{k}\mu_{k}\left(t\right)+p_{k}s_{k}\left(t\right)|S_{t}\right\}\left(A_{k}^{p}\left(t\right)+1\right)+E\left\{1-p_{k}\mu_{k}\left(t\right)-p_{k}s_{k}\left(t\right)|S_{t}\right\}\min\left\{A_{k}\left(t\right)+1,M\right\}. \end{array}$$

By substituting (A3) into (A2) and adding the term $VE\{c\left(t\right)|S_{t}\}$ on both sides, we obtain the result below

(A4) $$\begin{array}{cc} \; & \Delta \left(X\left(t\right)\right)+VE\left\{c\left(t\right)|S_{t}\right\}\leq \\ \; & \sum_{k=1}^{K}\frac{E\left\{\left(A_{k}^{2}\left(t+1\right)\right)|S_{t}\right\}+{\left(A_{k}^{\max}\right)}^{2}}{2}+\sum_{k=1}^{N}E\{X_{k}\left(t\right)[\left(A_{k}^{p}\left(t\right)+1\right)\left(p_{k}s_{k}\left(t\right)+p_{k}\mu_{k}\left(t\right)\right)\\ \; & +\min\left\{\left(A_{k}\left(t\right)+1\right),M\right\}\left(1-p_{k}s_{k}\left(t\right)-p_{k}\mu_{k}\left(t\right)\right)]|S_{t}\}-X_{k}\left(t\right)A_{k}^{\max}+VE\left\{c\left(t\right)|S_{t}\right\}. \end{array}$$

By setting $W_{k}\left(t\right)=p_{k}s_{k}\left(t\right)+p_{k}\mu_{k}\left(t\right)$, we obtain the result in (12).

## Appendix B. Proof of Theorem 2

**Proof.** Since the DPP algorithm uses the method of oppurtunistically minimizing an expectation, we obtain

(A5) $$\begin{array}{cc} \; & \Delta \left(X\left(t\right)\right)+VE\left\{c\left(t\right)|S_{t}\right\}\leq B+\sum_{k=1}^{K}X_{k}\left(t\right)E\{W_{k}\left(t\right)\left(A_{k}^{p}\left(t\right)+1\right)+\left(1-W_{k}\left(t\right)\right)\times \\ \; & \min\left[A_{k}\left(t\right)+1,M\right]\left|S_{t}\right\}-A_{k}^{\max}+VE\left\{c\left(t\right)|S_{t}\right\}\leq B+\sum_{k=1}^{K}X_{k}\left(t\right)E\left\{y_{k}^{*}\left(t\right)\right\}+VE\left\{c^{*}\left(t\right)\right\}, \end{array}$$

where $y^{*}\left(t\right)$ and $c^{*}\left(t\right)$ are the resulting vaues of ω policy as defined in Lemma 2. Note that the ω policy is independent of the system state $S_{t}$. By utilizing the bounds of Lemma 2, i.e., bound (14), we obtain By utilizing the bounds of Lemma 2, i.e., bound (14), we obtain

(A6) $$\begin{array}{c} \Delta \left(X\left(t\right)\right)+E\left\{c\left(t\right)|S\left(t\right)\right\}\leq B+\epsilon \sum_{k=1}^{K}X_{k}\left(t\right)+V\left(c^{\mathrm{opt}}+\epsilon \right), \end{array}$$

by taking ϵ→0, we have $\Delta \left(X\left(t\right)\right)+E\left\{c\left(t\right)|S\left(t\right)\right\}\leq B+Vc^{\mathrm{opt}},$ where is the exact form of the Lyapunov optimization theorem ([37] Theorem 4.2). Therefore, all virtual queues are mean-rate-stable, and therefore, the average constraints are satisfied. □

## Appendix C. Proof of Theorem 3

**Proof.** We apply the law of iterated expectations in Equation (A6), we obtain

(A7) $$\begin{array}{c} E\left\{L\left(X\left(t+1\right)\right)\right\}-E\left\{L\left(X\left(t\right)\right)\right\}+VE\left\{c\left(t\right)\right\}\leq B+\epsilon \sum_{k=1}^{K}X_{k}\left(t\right)+Vc^{\mathrm{opt}}+\epsilon . \end{array}$$

Summing over τ=0,1,2,…t−1, and diving by *t* and *V*, we obtain

$$\begin{array}{c} \sum_{\tau =0}^{t-1}\frac{1}{t}E\left\{c\left(t\right)\right\}\leq \frac{E\{L(X(t))\}-E\{L(X(0))\}}{Vt}+\frac{B}{V}+\frac{\epsilon }{V}\frac{1}{t}\sum_{k=1}^{K}X_{k}\left(t\right)+c^{\mathrm{opt}}+\frac{\epsilon }{V}. \end{array}$$

By taking ϵ→0, and t→∞, we obtain, $\lim_{t\to \infty }\sup\frac{1}{t}\sum_{\tau =0}^{t-1}E\left\{c\left(t\right)\right\}\leq \frac{B}{V}+c^{\mathrm{opt}}.$ This concludes the result. □
